# Lack of Fibronectin Extra Domain A Alternative Splicing Exacerbates Endothelial Dysfunction in Diabetes

**DOI:** 10.1038/srep37965

**Published:** 2016-11-29

**Authors:** Gianluca Gortan Cappellari, Rocco Barazzoni, Luigi Cattin, Andrés F. Muro, Michela Zanetti

**Affiliations:** 1 Department of Medical, Surgical and Health Sciences, University of Trieste, Trieste, Italy; 2 Mouse Molecular Genetics Laboratory, International Centre for Genetic Engineering and Biotechnology, Trieste, Italy

## Abstract

Glucose-induced changes of artery anatomy and function account for diabetic vascular complications, which heavily impact disease morbidity and mortality. Since fibronectin containing extra domain A (EDA + FN) is increased in diabetic vessels and participates to vascular remodeling, we wanted to elucidate whether and how EDA + FN is implicated in diabetes-induced endothelial dysfunction using isometric-tension recording in a murine model of diabetes. In thoracic aortas of EDA^−/−^, EDA^+/+^ (constitutively lacking and expressing EDA + FN respectively), and of wild-type mice (EDA^wt/wt^), streptozotocin (STZ)-induced diabetes impaired endothelial vasodilation to acetylcholine, irrespective of genotype. However STZ + EDA^−/−^ mice exhibited increased endothelial dysfunction compared with STZ + EDA^+/+^ and with STZ + EDA^wt/wt^. Analysis of the underlying mechanisms revealed that STZ + EDA^−/−^ mice show increased oxidative stress as demonstrated by enhanced aortic superoxide anion, nitrotyrosine levels and expression of NADPH oxidase NOX4 and TGF-β1, the last two being reverted by treatment with the antioxidant n-acetylcysteine. In contrast, NOX1 expression and antioxidant potential were similar in aortas from the three genotypes. Interestingly, reduced eNOS expression in STZ + EDA^+/+^ vessels is counteracted by increased eNOS coupling and function. Although EDA + FN participates to vascular remodelling, these findings show that it plays a crucial role in limiting diabetic endothelial dysfunction by preventing vascular oxidative stress.

Diabetes-associated vascular disease is characterized by endothelial dysfunction and by excess accumulation of basement membrane proteins in the subendothelial layer^1^,^2^. Fibronectin (FN), a multidomain glycoprotein classically involved in the regulation of cell-cell and cell–matrix interactions, has an essential role in diabetic angiopathy^3^,^4^. FN expression is subject to alternative splicing of the primary transcript at three sites: the cassette-type extra domains A (EDA or EIIIA) and B (EDB or EIIIB), and the type III connecting segment, generating up to 20 different isoforms^5^,^6^. Unlike for EDB + FN, several specific functions for EDA + FN in adult subjects have recently been described, mainly related to wound healing, tissue fibrosis, thrombosis and vascular wall integrity^5^,^6^,^7^. EDA + FN inclusion is highly restricted in adult healthy vessels but increases in vascular disease and in atherosclerosis^8^. In this setting, it contributes to plaque enlargement but also to its stabilization^9^,^10^. In addition, reduced expression of EDA + FN has been associated with thoracic aortic aneurysm formation^11^ as well as with hemorrhage of the vessel wall^12^. Finally, mice strains devoid of EDA exon regulated splicing show reduced plaque formation, indicating EDA splicing as an important broad modulator of atherogenesis^13^,^14^.

Diabetes, a well-known risk factor of vascular disease, is associated with enhanced vascular synthesis, plasma levels and vascular deposition of EDA- and EDB- expressing FN^15^,^16^. These events, which characterize diabetic angiopathy, initiate important events in response to injury^17^. Several studies have shown that transforming growth factor beta (TGF-β), typically activated in diabetic vessels^18^, is a potent inducer of extracellular matrix deposition and of EDA + FN splicing^19^,^20^.

Endothelial dysfunction, characterized by increased superoxide production, reduced nitric oxide (NO) bioavailability and impaired endothelium-dependent dilation, is a hallmark of diabetes^1^. Hyperglycemia enhances NADPH oxidase activity to generate superoxide^21^, which combines with nitric oxide, to form peroxynitrite. Peroxynitrite rapidly oxidizes the eNOS cofactor tetrahydrobiopterin, determining eNOS uncoupling^22^,^23^,^24^. In these conditions, the enzyme actively produces superoxide rather than NO, further contributing to oxidative stress and endothelial dysfunction. In addition, NADPH oxidase-induced eNOS uncoupling mediates extracellular matrix injury and stimulates de-novo FN synthesis^24^, which in turn negatively modulates eNOS expression and activity in endothelial cells^25^. The interaction between EDA + FN, reactive oxygen species (ROS) and eNOS is currently unclear and the potential functional role of EDA + FN accumulation in the setting of diabetes-associated endothelial dysfunction is unknown.

## Results

### Animal characteristics, survival, hormonal and metabolic profile

To evaluate the impact of EDA + FN alternative splicing on endothelial function during diabetes, we used male mice from two engineered mouse strains^26^ lacking regulated splicing of fibronectin EDA exon, one constitutively including (EDA^+/+^) and the other constitutively excluding the exon (EDA^−/−^), due to the optimization of the EDA splicing sites and to the targeted deletion of the EDA exon, respectively. Baseline body weight and glucose were comparable in EDA^+/+^, EDA^−/−^ and EDA^wt/wt^ mice. Basal insulin levels tended to be higher (p < 0.05) in EDA^wt/wt^, while still in physiological range^27^ (Table 1). STZ treatment resulted in sustained blood glucose concentration of >18 mmol/liter in >90% of mice without differences among genotypes. At 16 weeks after treatment, STZ-induced diabetic mice showed reduced body weight and plasma insulin compared to control (Table 1).

### Constitutive exclusion of EDA domain increases endothelial dysfunction to acetylcholine in STZ-treated mice without modifying endothelium-independent dilation to DEA-NONOate

**Figure 1:**
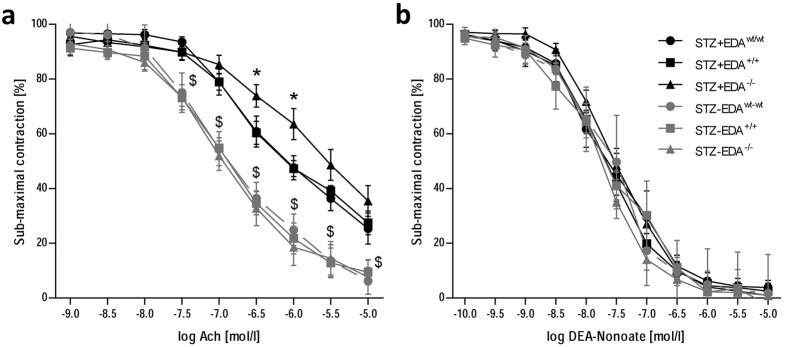
(**a**) Endothelium-dependent vasodilation to acetylcholine is impaired in diabetic (STZ+) EDA^−/−^ mice. Diabetes reduced endothelium-dependent dilation in all animal genotypes, however, STZ + EDA^−/−^ mice showed increased endothelial dysfunction compared with STZ + EDA^wt/wt^ and STZ + EDA^+/+^. In contrast endothelium-independent vasodilation to the NO donor DEA-NONOate (**b**) was similar in control (STZ-) and diabetic EDA^wt/wt^, EDA^+/+^ and EDA^−/−^ mice suggesting preserved arterial smooth muscle function. Data represent at least 7 mice per group. STZ: streptozotocin; ^$^p < 0.05 non diabetic vs. STZ; *p < 0.05 STZ + EDA^−/−^ vs. STZ + EDA^wt/wt^ and STZ + EDA^+/+^.

Isometric tension recording studies in organ chambers demonstrated that submaximal contractions to phenylephrine were similar in aortas from diabetic and non-diabetic EDA^wt/wt^, EDA^+/+^ and EDA^−/−^ mice. Aortas from non-diabetic animals showed similar relaxations to acetylcholine (Fig. 1a). STZ treatment resulted in impaired endothelium-dependent dilation to acetylcholine in all animal genotypes as expected (Fig. 1a). However, STZ + EDA^−/−^ mice showed increased (p < 0.05) endothelial dysfunction compared with STZ + EDA^wt/wt^ and STZ + EDA^+/+^ (Fig. 1a), suggesting reduced NO bioavailability. Endothelium-independent vasodilation to DEA-NONOate did not differ among groups, both under euglycemic and hyperglycemic conditions (Fig. 1b).

### Total fibronectin levels and eNOS expression and coupling state are preserved in EDA^−/−^ diabetic aorta

**Figure 2:**
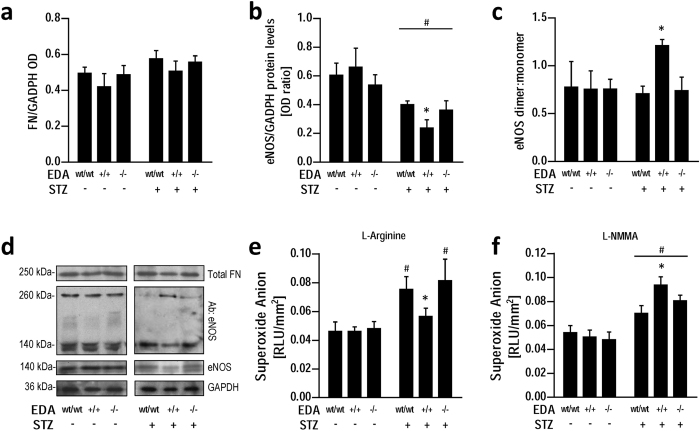
Expression of total fibronectin and protein levels, coupling state and function of eNOS in aortas from diabetic (STZ+) EDA^−/−^, EDA^+/+^ and EDA^wt/wt^ compared with control (STZ-) mice. Relative protein expression of total fibronectin (**a**), eNOS (**b**) and eNOS dimer to monomer ratio (**c**), with representative blots (**d**). Superoxide generation in aortic rings of control and diabetic mice in the presence of either the NOS substrate L-arginine (1 mmol/l) (**e**) or the enzyme inhibitor L-NMMA (100 μmol/l) (**f**). Data are normalized to parietal surface and represent at least 7 mice per group. STZ: streptozotocin; OD: Optical Density; RLU: relative light units. *p < 0.05 vs other groups with same STZ treatment; #p < 0.05 vs. STZ- groups.

Total fibronectin levels were comparable between aortas within both control and diabetic genotypes, with STZ treated groups showing a trend for increased protein deposition, in agreement with reports in humans and in rodent diabetic models^28^,^29^ (Fig. 2a and d). To assess whether endothelial dysfunction in STZ + EDA^−/−^ mice was related to decreased eNOS expression, total eNOS protein levels were measured in aortas from control and diabetic mice. In our model, STZ was associated with lower total eNOS protein levels^30^ in all genotypes with neither significant differences among genotypes of the non-diabetic control animals, nor between STZ + EDA^−/−^ and STZ + EDA^wt/wt^ samples (Fig. 2b and d). Accordingly, eNOS dimer/monomer ratio, an accepted marker of eNOS uncoupling and function, was similar in EDA^−/−^ and in EDA^wt/wt^ both in control and diabetic conditions (Fig. 2c and d). Unexpectedly, during diabetes eNOS protein further decreased by 60% in STZ + EDA^+/+^ compared with STZ + EDA^−/−^ and STZ + EDA^wt/wt^ (p < 0.05) (Fig. 2b and d). However, this change was compensated by increased (p < 0.05) enzyme coupling, as demonstrated by higher eNOS dimer/monomer ratio in this group (Fig. 2c and d). To confirm these data, we performed analysis of eNOS-derived superoxide in vessels from control and diabetic mice. Incubation of aortic rings with L-arginine, a substrate of NOS, as well as with the competitive inhibitor L-NMMA, did not produce different effects on superoxide generation in aortic rings among control groups and in STZ + EDA^−/−^ compared to STZ + EDA^wt/wt^ mice (Fig. 2e and f). Instead, in STZ + EDA^+/+^ mice superoxide generation was lowered (p < 0.05) by L-arginine and increased (p < 0.05) by L-NMMA treatments (p < 0.05), confirming increased (p < 0.05) eNOS coupling state in this group (Fig. 2e and f). These data support the hypothesis of a compensatory mechanism resulting in similar NO production, functionally confirmed by the lack of differences in endothelial function between diabetic EDA^+/+^ and EDA^wt/wt^ groups (Fig. 1a).

### Increased endothelial dysfunction in STZ + EDA^−/−^ mice is associated with increased vascular ROS and nitrotyrosine generation in the presence of preserved antioxidant potential

**Figure 3:**
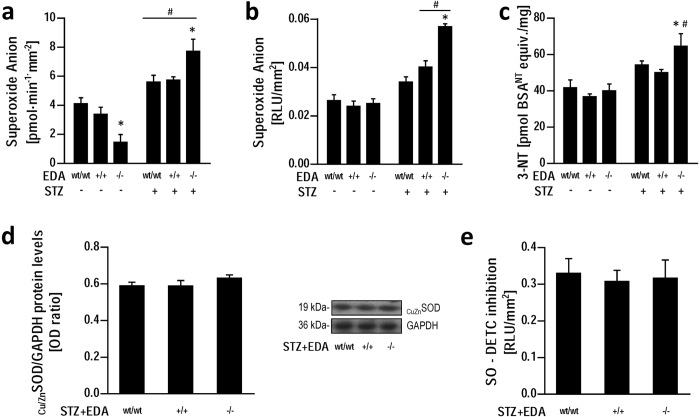
Vascular oxidative stress is increased in aortas from diabetic (STZ+) EDA^−/−^ mice with unchanged antioxidant potential. Quantitive analysis of aortic superoxide production by cytochrome c reduction (**a**) and by lucigenin-enhanced chemiluminescence (**b**); 3-nitrotyrosine levels in aortas from control (STZ−) and diabetic (STZ+) mice aortas (**c**). Relative expression of the antioxidant enzyme CuZnSOD with representative blot (**d**); effect of the SOD inhibitor DETC (100 μmol/l) on superoxide production in presence of NADPH (100 μmol/l) (**e**). Data are normalized to parietal surface and represent at least 7 mice per group. a.u.: arbitrary units; 3-NT: 3-Nitrotyrosine; BSA: Bovine Serum Albumin; RLU: relative light units; STZ: streptozotocin; SO: Superoxide Anion. *p < 0.05 vs other groups with same STZ treatment; #p < 0.05 vs. STZ- groups.

We then focused our attention on dissecting the mechanisms underlying endothelial dysfunction in STZ + EDA^−/−^ mice by investigating ROS generation and redox balance. Aortic ROS generation was directly assessed by measuring tissue superoxide production using SOD-inhibitable cytochrome c reduction and lucigenin-enhanced chemiluminescence. In control animals superoxide production as assessed by cytochrome c reduction was lower (p < 0.05) in aortas from EDA^−/−^ mice, however these data were not confirmed by lucigenin-enhanced chemiluminescence (Fig. 3a and b). Importantly, during diabetes superoxide production increased (p < 0.05) in the three genotypes according to both methods (Fig. 3a and b). However, STZ + EDA^−/−^ demonstrated higher vascular superoxide production than STZ + EDA^wt/wt^ and STZ + EDA^+/+^ (Fig. 3a and b). Protein tyrosine nitration, resulting from superoxide combining with NO was also measured. STZ + EDA^−/−^ exhibited the highest 3-nitrotyrosine concentrations among groups (Fig. 3c).

To verify that these findings were due to enhanced superoxide production rather than reduced antioxidant potential, antioxidant expression and activity were measured in aortas from experimental animals. There were no differences in aortic protein expression of copper/zinc superoxide dismutase (_CuZn_SOD) among the different genotypes of STZ-treated mice (Fig. 3d). After the addition of the substrate NADPH and blockade with DETC, inhibition of superoxide production was similar in aortic rings from all of the STZ experimental groups (Fig. 3e), demonstrating similar SOD potential.

### Constitutive exclusion of EDA domain increases NADPH oxidase activity selectively upregulating NOX4 expression in diabetic mice aorta

**Figure 4:**
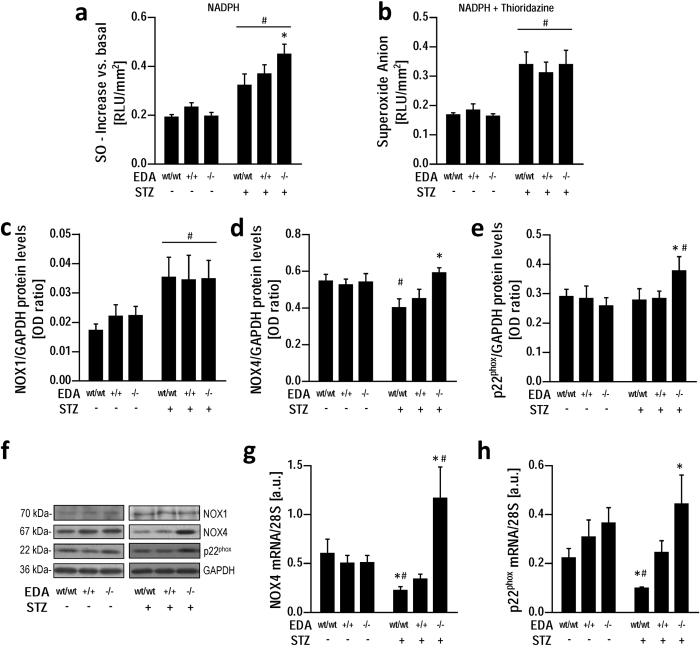
NADPH oxidase activity is increased and NADPH oxidase isoform NOX4 expression is selectively upregulated in diabetic (STZ+) aortas from EDA^−/−^ mice. After stimulation with NADPH (100 μmol/l) superoxide production increased in aortas from STZ + EDA^−/−^ mice (**a**) and was normalized by incubation with the NADPH oxidase inhibitor thioridazine (10 μmol/l), also in the presence of NADPH (100 μmol/l) (**b**). Densitometric analysis of NOX1 (**c**), NOX4 (**d**) and p22^phox^ (**e**) protein expression in STZ- and STZ+ treated groups; representative Western blots in control and diabetic groups (**f**); NOX4 mRNA (**g**) and p22^phox^ mRNA (**h**) in the same groups. Data represent at least 7 mice per group. *Ex-vivo* functional measurements are normalized to parietal surface. RLU: Relative Light Units; STZ: Streptozotocin; OD: Optical Density; SO: Superoxide Anion; a.u.: arbitrary units; *p < 0.05 vs other groups with same STZ treatment; #p < 0.05 vs. STZ- groups.

To further investigate the differences in vascular ROS generation induced by the constitutive exclusion of the EDA exon in EDA^−/−^ diabetic mice, aortic superoxide anion generation was measured in the presence of substrates or inhibitors of NADPH oxidase, which is notably a crucial source of vascular ROS in diabetes. After incubation of aortic rings with NADPH, vascular superoxide production increased (p < 0.05) in STZ-treated animals compared with respective control groups (Fig. 4a). However, the increase was more pronounced (p < 0.05) in aortas from STZ + EDA^−/−^ mice suggesting an involvement of NADPH oxidase in the higher ROS production observed in this group. This finding was confirmed by normalization of superoxide anion generation in STZ + EDA^−/−^ after inhibition of NADPH oxidase by thioridazine, also in the presence of the substrate NADPH (Fig. 4b). To further characterize the contribution of NADPH oxidase to ROS generation, we investigated the expression of isoforms NOX1 and NOX4. In aortas from wild type mice STZ treatment increased NOX1 but not NOX4 protein expression (Fig. 4c,d and f). However, among diabetic animals, while NOX1 protein expression was not different among genotypes (Fig. 4c and f), both NOX4 protein and gene expression selectively increased (p < 0.05) in STZ + EDA^−/−^ compared to STZ + EDA^wt/wt^ and STZ + EDA^+/+^ (Fig. 4d,f and g). Similar effects were detected for the modulatory subunit p22^phox^ (Fig. 4e,f and h).

### NOX4 is associated with increased TGF-β1 expression but not PKC activation in EDA^−/−^ diabetic aorta, and is reversed by exogenous antioxidant administration

**Figure 5:**
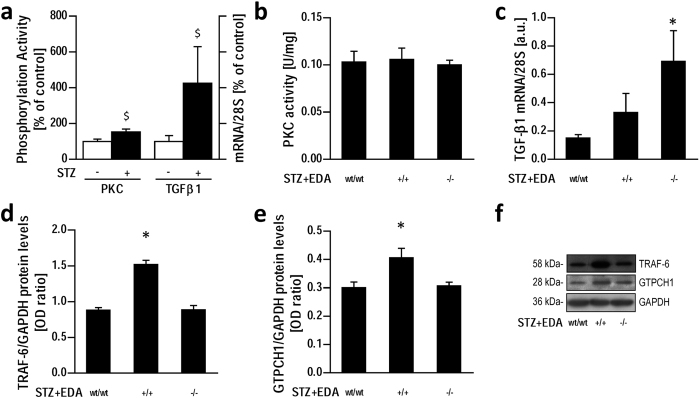
Effect of diabetes on protein kinase C and TGF-β1 in aortas from experimental animals. Protein Kinase C (PKC) activity and TGF-β1 mRNA relative increase in the aortas of wild type mice after STZ treatment (**a**); PKC activity (**b**) and TGF-β1 mRNA levels in diabetic groups (**c**). Densitometric analysis of TRAF-6 (**d**) and GTPCH1 (**e**) protein expression in STZ-treated groups with representative blots (**f**). Data represent at least 7 mice per group. STZ: Streptozotocin; OD: Optical Density; a.u.: arbitrary units; *p < 0.05; ^$^p < 0.05 vs respective STZ- group.

**Figure 6:**
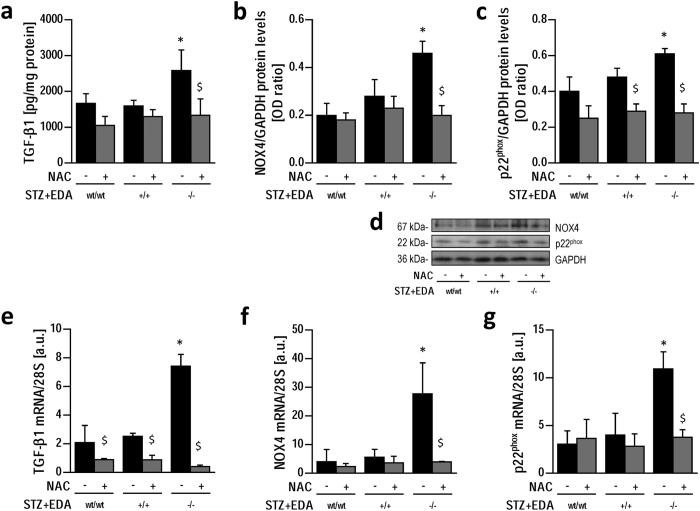
Impact of antioxidant treatment with n-acetylcysteine (NAC) on aortic TGF-β1 (**a**), NOX4 (**b**) and p22^phox^ (**c**) protein levels, with representative blots (**d**). TGF-β1 (**e**), NOX4 (**f**) and p22^phox^ (**g**) mRNA levels in the same groups. Data represent at least 5 mice per group. OD: Optical Density; a.u.: arbitrary units; STZ: streptozotocin; *p < 0.05 vs. other groups; ^$^p < 0.05 vs. respective NAC- group.

Since both protein kinase C (PKC) and TGF-β1 pathways are known activators of NOX4 and play a role in the pathogenesis of diabetic vascular complications^31^,^32^, we explored their potential involvement in the selective upregulation of NOX4 in STZ + EDA^−/−^ aortas. As expected, STZ treatment resulted in increased (p < 0.05) PKC activity and TGF-β1 gene and protein expression in aortas from wild type mice (Fig. 5a), but while in diabetic mice PKC activity was similar among all genotypes (Fig. 5b), TGF-β1 gene expression increased (p < 0.05) in STZ + EDA^−/−^ mice aortas compared to both STZ + EDA^wt/wt^ and STZ + EDA^+/+^ (Fig. 5c). In a further set of experiments this change was prevented, also at protein expression level, by treatment for 12 weeks with the antioxidant n-acetylcysteine (NAC, Fig. 6a and e), indicating that oxidative stress is a major drive in diabetes-induced TGF-β1 upregulation in STZ + EDA^−/−^ mice. Furthermore, NAC treatment normalized also NOX4 and p22^phox^ mRNA and protein levels in aortas from diabetic mice, resulting in the lack of differences among genotypes (Fig. 6b–d and f,g).

### TRAF-6 expression is associated with eNOS modulation but not with TGF-β1 or NOX4 expression in EDA^−/−^ diabetic aorta

Since EDA is a major endogenous ligand for TLR-4 we finally evaluated the protein expression levels of TRAF-6, an important TLR-4 classical signaling pathway mediator known to induce NF-κB activation^33^. Western blot analysis of diabetic aortas showed increased TRAF-6 expression in diabetic EDA^+/+^ mice (Fig. 5d and f). Moreover, this finding was associated with the upregulation of GTPCH1 expression, the main enzyme involved in the synthesis of the NOS coupling factor BH_4_ (Fig. 5e and f), known to be increased after TLR-4-triggered NF-κB activity^34^,^35^. However, no differences in both TRAF-6 and GTPCH1 expression levels were detected in STZ + EDA^−/−^ compared to wild type (Fig. 5d–f).

## Discussion

Impaired endothelium-dependent vasodilation and structural remodelling of the vessel wall, i.e. thickening of the subendothelial layer and accumulation of extracellular matrix proteins, are known features of diabetic vascular disease^1^,^36^. The fibronectin isoform containing the alternatively spliced EDA domain is normally absent from the circulation, but elevated plasma levels of EDA+ FN are found in diabetic patients^16^ and in other pathological conditions involving vascular disease^37^,^38^,^39^,^40^,^41^. Regulation of alternative splicing to generate the EDA + FN isoform is part of the response to hyperglycemia, and this appears to be potentially relevant and of great interest, as specific functions have been progressively identified for this domain^4^,^11^,^12^,^13^. However how EDA + FN might contribute to the modulation of endothelial dysfunction in diabetes is unclear.

This study provides evidence for a role of the EDA domain of FN in diabetes-associated endothelial dysfunction. STZ-induced diabetic mice devoid of the EDA exon (EDA^−/−^ mice) showed increased endothelial dysfunction, suggesting a potential protective role of EDA + FN in the pathogenesis of the disease. Further experiments to identify the underlying mechanisms demonstrated that EDA + FN reduces vascular oxidative stress and prevents eNOS uncoupling. To date, no interactions of EDA + FN with cellular pathways of endothelial dysfunction in diabetes have been described.

Vascular exposure to high glucose results in increased superoxide production and oxidative stress via vascular NADPH oxidase activation, mediated by the upregulation of the NOX1 and NOX4 isoforms^42^,^43^,^44^, which play a role in the development of diabetes-associated endothelial dysfunction^22^. However the contribution of NOX1 to increased superoxide production and oxidative stress appears prominent compared to that of NOX4 during diabetes^43^. In fact, apparently vascular NOX4 is not directly involved in diabetes-associated endothelial dysfunction^43^, but it may have indirect effects through protein-protein interactions and regulation of peroxynitrate formation^23^,^24^. Consistently with previous findings^43^, we found increased NOX1 expression after induction of diabetes with STZ in all genotypes, while NOX4 selectively increased only in EDA^−/−^ diabetic mice. Accordingly, vascular superoxide generation and levels of oxidative stress marker nitrotyrosine were higher in aortas from diabetic animals than in control group, but diabetic EDA^−/−^ mice displayed the highest nitrotyrosine levels, a finding compatible with the highest NOX4 expression in this group^24^,^42^. The reason(s) for overexpression of NOX4 in STZ + EDA^−/−^ diabetic aortas are largely unknown and, to date, no direct interaction between EDA and NOX4 has been demonstrated. Moreover, the finding of similar levels of NOX4 expression and activity in non-diabetic mice displaying different genotypes suggests that NOX4 overexpression during diabetes is selectively linked to the lack of EDA domain. Mechanisms underlying diabetes-induced NADPH oxidase upregulation are complex and include the activation of signaling pathways such as protein kinase C and TGFβ^44^,^45^. Altogether, these pathways promote extracellular matrix accumulation and the development of vascular complications in diabetes^31^,^46^,^47^. In our study PKC was similarly activated in all diabetic genotypes, excluding, therefore, its implication in NOX4 overexpression in vessels from diabetic EDA^−/−^ mice. In contrast, TGFβ1 mRNA was higher in EDA^−/−^ compared with EDA^+/+^ and EDA^wt/wt^ diabetic aortas. TGFβ selectively increases NOX4 (but not NOX1) expression and activity in several cell types, including fibroblasts, smooth muscle cells and liver stellate cells^44^. Treatment of experimental animals with the non-selective antioxidant n-acetylcysteine abrogated TGFβ, NOX4 and p22^phox^ upregulation. This finding is in line with recent studies, demonstrating that either ROS scavengers or selective inhibition of NOX4 modulate TGFβ activity^48^. The mechanisms underlying TGFβ1 regulation by EDA+FN are currently largely unknown. Since EDA is a well-known endogenous ligand of TLR4^4^,^8^, one hypothesis is that TLR4 pathway might be involved in the observed results. Our findings show increased NOX4 expression and activity in the aortas of mice lacking EDA expression, suggesting for this finding a mechanism independent of NOX4 activation by direct interaction with TLR-4 as described by Park *et al.*^49^ Moreover, in our study we found that the expression of TRAF-6, an important TLR-4 signalling pathway mediator, was not modified in the aorta of diabetic EDA^−/−^ compared to wild type, suggesting that also classical TLR-4 signalling via TRAF6 is not directly involved in the increase of reactive oxygen species observed in that group. Other mechanisms might therefore be implicated, potentially including the involvement of alternative modulatory pathways in the crosstalk between TLR-4 and TGFβ systems, as suggested by reports showing that TLR-4 activation downregulates TGFβ signaling via Smads in the microglia^50^. Moreover, since TGFβ1 affects FN splicing by favoring the inclusion of EDA^11^,^20^, our data consistently support the possibility of a negative feedback loop between EDA + FN, TGFβ1 and NOX4 in diabetes, as previously suggested by Kawelke *et al.*^51^ in liver fibrosis between FN and TGFβ. In this setting, adequate amounts of EDA + FN are required to limit TGFβ1 overexpression and activation^51^. In addition, reduced stimulation of TLR4 in EDA^−/−^ mice resembles a situation similar to that described in TLR4^−/−^ mice, also presenting endothelial dysfunction via upregulation of NADPH oxidase^52^,^53^. Further experiments will be required to better elucidate the complex network interplay which appear to underlie the effect of EDA on ROS production in diabetes.

Increased ROS formation by glucose-stimulated NADPH oxidase activation may either upregulate or downregulate antioxidant enzymes^54^. In our study, however, antioxidant activity did not differ among diabetic animals, suggesting that upregulation of NADPH oxidase in EDA^−/−^ diabetic mice is selectively related to ROS formation and not to their scavenging via antioxidant mechanisms. Similarly, STZ-induced diabetes did not alter total eNOS protein in aorta in WT and EDA^−/−^ mice. However, eNOS expression was decreased in EDA^+/+^ diabetic aortas, suggesting that EDA + FN, an isoform bearing a conformation that better exposes the RGD cell binding site recognized by the α_5_β_1_ integrin^7^, is more active in down-regulating eNOS gene expression than FN lacking the EDA domain. This hypothesis and our current findings are in agreement with previous results showing that plasma FN (which lacks the EDA domain) modulates eNOS gene and protein expression in a α_5_β_1_ integrin-Akt/p38/MAP kinase dependent pathway in endothelial cells^25^, suggesting that EDA domain might be specifically involved in this process. Overproduction of superoxide anion reduces NO bioavailability by direct scavenging. In addition to NADPH oxidase, uncoupled eNOS is also a relevant source of superoxide in diabetic vessels^2^. By using L-NMMA-inhibitable superoxide formation to evaluate the contribution of uncoupled eNOS to total superoxide production we found that enzyme uncoupling did not contribute to ROS overproduction in diabetic aortas from either genotype; instead, a reduced enzyme uncoupling in aortas of STZ -EDA^+/+^ mice was suggested from available data. These results were confirmed by protein analysis, which showed that, in spite of reduced total eNOS protein, eNOS dimerization was increased in vessels from STZ + EDA^+/+^. These data, together with the observation that endothelial damage in STZ + EDA^+/+^ animals was similar to that of STZ + EDA^wt/wt^ mice, suggest that NO bioactivity in terms of NO and superoxide production is not fully reflected by levels of eNOS protein expression in these mice, as previously reported in diabetes^22^,^55^. Indeed, NADPH-derived superoxide can oxidize BH_4_ under physiological conditions and induce eNOS uncoupling^24^,^56^. However, diabetic uncoupling of eNOS due to NADPH-derived superoxide is mediated by NOX1^57^, which was similarly activated in STZ + EDA^−/−^ and in STZ + EDA^+/+^. Therefore, it is unlikely that this pathway is involved in the different enzyme coupling state observed among diabetic genotypes. Our data instead provide a mechanistic insight by showing that STZ + EDA^+/+^ mice present increased protein levels of the TLR-4 signalling mediator TRAF-6 in association with the upregulation of GTPCH1, the main enzyme involved in the synthesis of the NOS coupling factor BH_4_. Importantly, GTPCH1 expression is known to be increased in endothelial cells by enhanced NF-κB activity, a main target activated by TRAF-6 signaling^33^,^35^. This pathway linking TLR-4 activation with enhanced GTPCH1 expression has been recently fully described in macrophages^34^. While this mechanism remains mainly speculative until further investigation, these data are in excellent agreement with the hypothesis of EDA as a modulator of eNOS coupling in diabetic aorta via TLR-4.

Collectively, we showed a novel modulatory mechanism for endothelial dysfunction in diabetes. Lack of EDA + FN worsens endothelial function by increasing NADPH-oxidase NOX4 derived ROS production and TGF-β1 expression. Conversely, EDA + FN lowers eNOS expression but, at the same time, compensates its potential lower activity by preventing eNOS uncoupling. Therefore, while on one side EDA + FN expands the extracellular matrix resulting in diabetic structural vascular abnormalities, on the other side it prevents endothelial dysfunction by providing a feedback defence against excessive ROS generation. Modulation of FN alternative splicing might impact the course of vascular disease in diabetes.

## Materials and Methods

### Ethics statement

All experiments were performed in accordance with relevant guidelines and regulations issued by the European Parliament (Directive 2010/63/EU for animal experimentation). All experimental protocols were approved by the board of the International Centre for Genetic Engineering and Biotechnology where the study was conducted.

### Animals and Induction of Diabetes

We used male mice from two engineered mouse strains previously generated in our laboratory: the EDA^+/+^ strain, constitutively including the EDA exon and the EDA^−/−^ strain, constitutively excluding the EDA exon^26^. Mouse strains were backcrossed with C57Bl/6 wild type (EDA^wt/wt^) for at least 6 generations. Diabetes was induced in mice, aged 12 weeks (n = 43 EDA^wt/wt^; n = 44 EDA^+/+^; n = 40 EDA^−/−^) by intraperitoneal injection of streptozotocin (STZ, 50 μg/kg body weight/day freshly dissolved in 0.1 M citrate buffer, pH 4.5, delivered for five consecutive days). Control mice were vehicle injected (n = 26 EDA^wt/wt^; n = 30 EDA^+/+^; n = 28 EDA^−/−^). A further subset of mice from each genotype was treated with STZ and the antioxidant N-acetylcysteine (NAC; 1.5 g kg^−1^ day^−1^ dissolved in drinking water^58^) was administered from week 5 of diabetes (n = 10 NAC-EDA^wt/wt^; n = 9 NAC-EDA^+/+^; n = 11 NAC-EDA^−/−^). Diabetes onset was confirmed by blood glucose levels >250 mg/dL (11 mmol/L) one week after the first dose of STZ using blood drawn by the tail vein. Animals not responding to STZ treatment were excluded. Mice were inspected daily, 5 h fasting blood glucose concentrations and body weight of STZ-treated animals were measured before treatment, one week later and then on a monthly basis. Control animals were measured before treatment and after 12 and 16 weeks. After 16 weeks of diabetes, animals were anesthetized by intraperitoneal injection of avertin (20 μl/g body weight, 1.25% solution in PBS), blood was collected through cardiac puncture and aortas were harvested, cleaned of connective tissue and either used for analytical determinations or immediately frozen.

### Hormonal and Metabolic Profile

Blood glucose was measured by reflectometry (AccuCheck, Roche, Indianapolis, IN, USA). Plasma lipids were measured by standard commercial colorimetric enzymatic assays (BioMerieux, Marcy l’Etoile, France; Roche Diagnostics, Basel, Switzerland). Plasma insulin concentration was determined using a commercially available ELISA kit (DRG International Mountainside, NJ, USA).

### Analysis of Vascular Reactivity

Isometric tension of mouse aorta was measured *ex vivo* in isolated organ baths, as previously described^59^,^60^. Briefly, rings (3 mm long) from each thoracic aorta were used for assessing vascular reactivity immediately after dissection. Rings were suspended in organ chambers filled with 25 ml of gassed (95% O_2_ and 5% CO_2_) modified Krebs-Ringer bicarbonate solution (composition in mmol L-1: 118.3 NaCl, 4.7 KCl, 2.5 CaCl_2_,1.2 MgSO_4_, 1.2 KH_2_PO_4_, 25.0 NaHCO_3_, 0.026 EDTA, 11.1 dextrose, pH 7.4). The rings were allowed to equilibrate for 1 h at 37 °C and then stretched to the optimal point as determined by repeated exposure to 20 mmolˑL^−1^ KCl. The maximal contraction of each ring was determined by phenylephrine (PHE) 10^−5^ molˑL^−1^. After washing and re-equilibration, submaximal contraction was then obtained using a 10^−6^ molˑL^−1^ concentration of PHE. Dose-relaxation curves were finally performed by subsequent cumulative addition of acetylcholine (10^−9^ to 10^−5^ molˑL^−1^) or DEA-NONOate (2-(N,N-diethylamino)-diazenolate-2-oxide; 10^−10^ to 10^−5^ molˑL^−1^) to the precontracted rings.

### Protein expression analysis by Western Blot and ELISA

Proteins were extracted from the Tri-Reagent homogenates described below, after RNA extraction. Following ethanol mediated DNA precipitation and removal, proteins were collected by acetone precipitation, washed thrice with guanidinium hydrochloride solution (0.5 mol/l in 95% w/v ethanol), once with absolute ethanol and finally resuspended in SDS solution (1% w/v in ultrapure water) with protease inhibitors (P8340, Sigma, St.Louis, MI, USA). Samples were separated by SDS-PAGE and Western blot analysis performed as previously described^59^,^60^. Equal load was checked by Ponceau S staining and GAPDH probing. Antibodies used in the present study as well as their dilution are described in Supplemental Table 1. Results from densitometric analyses are expressed as ratio between target protein and GAPDH optical densities. Low-temperature electrophoresis on 5% polyacrylamide gel (LT-PAGE) was performed for detection of eNOS dimer as reported^22^. Protein levels of TGF-β1 were assessed by ELISA (Abcam) according to manufacturer’s recommendations.

### Assessment of systemic and vascular redox state

Superoxide anion in intact aortic rings was measured by the SOD-inhibitable cytochrome c reduction assay as described^59^ and by low concentration (10 μmol/l) lucigenin-enhanced chemiluminescence. eNOS- and NADPH oxidase derived superoxide and SOD activity in aortic rings were measured by addition of the following substrates or inhibitors: L-arginine (1 mmol/l), L-NMMA (100 μmol/l), thioridazine (10 μmol/l), diethyldithiocarbamate (DETC: 100 μmol/l) and NADPH (100 μmol/l). Data from incubated aortic rings was normalized by endothelial surface as measured (ImageJ) on a high-resolution scan of the opened vessel rings. Nitration of protein tyrosine in aortic tissue samples was measured by chemiluminescence enhanced indirect ELISA, as previously published^59^.

### Quantitative Real Time PCR

Aortas underwent mechanical homogenization in Tri-Reagent (Sichim, Rome, Italy) followed by chloroform extraction and RNA precipitation in cold isopropanol. The resulting pellet was then washed with ethanol 70% (v/w) and resuspended in water. The purity of extracted RNA was checked by spectrophotometric determination (Nanodrop, Thermo Scientific, Wilmington, DE, USA) and its quality was verified in randomly selected samples by on-chip electrophoresis (2100 Bioanalyzer, Agilent, Santa Clara, CA, USA). All samples were treated with DNAse I (1 U/μg RNA; Boehringer Mannheim, Mannheim, Germany) and reverse transcribed to cDNA using a commercially available kit (Taqman Reverse Transcription Reagents, Applied Biosystems). Quantitative real time polymerase chain reaction was performed on a real time scanner (ABI prism 7900HT, Applied Biosystems) using TaqMan technology with custom designed primers and probes (Applied Biosystems; Eurofins MWG Operon). Primer and probe sequences used are shown in Supplemental Table 2. Relative quantification of gene expression was performed by interpolation of results on a standard curve of serial dilutions of a pool of samples and normalized by 28 S expression.

### Protein kinase C activity

Protein kinase C activity in aortic rings was measured by a commercially available non-radioactive assay of peptidic phosphorylation (PepTag PKC assay, Promega, Madison, WI, USA) following manufacturer’s instructions.

### Statistical Analysis

Statistical analysis was performed with SPSS (v.17) using t-test or ANOVA followed by post-hoc tests, for unpaired or for repeated measures as appropriate. Results from multiple comparisons were corrected according to Benjamini-Hochberg. Quantitative data are presented as the mean ± S.E. A p value < 0.05 was considered statistically significant.

## Additional Information

**How to cite this article**: Gortan Cappellari, G. *et al.* Lack of Fibronectin Extra Domain A Alternative Splicing Exacerbates Endothelial Dysfunction in Diabetes. *Sci. Rep.* **6**, 37965; doi: 10.1038/srep37965 (2016).

**Publisher's note:** Springer Nature remains neutral with regard to jurisdictional claims in published maps and institutional affiliations.

## Supplementary Material

Supplementary Information

## Figures and Tables

**Table 1:** Animal phenotype.

	Control			STZ		
	EDA^wt/wt^	EDA^+/+^	EDA^−/−^	EDA^wt/wt^	EDA^+/+^	EDA^−/−^
n	26	30	28	43	44	40
Body weight (g)						
Week 0	27.5 ± 0.8^a^	27.4 ± 0.6^a^	26.8 ± 0.8^a^	27.6 ± 0.5^a^	25.9 ± 1.0^a^	26.1 ± 0.4^a^
Week 16	35.2 ± 0.7^a^*	34.4 ± 0.4^a^*	33.3 ± 0.7^a^*	28.6 ± 0.6^b^	29.5 ± 0.8^b^	26.6 ± 0.5^c^
Blood glucose (mmol/l)						
Week 0	8.0 ± 0.3^a^	8.4 ± 0.2^a^	8.3 ± 0.4^a^	7.8 ± 0.3^a^	8.4 ± 0.4^a^	8.4 ± 0.4^a^
Week 16	8.3 ± 0.3^a^	9.1 ± 0.3^a^	9.0 ± 0.3^a^	24.7 ± 0.9^b^*	23.3 ± 1.1^b^*	23.0 ± 1.6^b^*
Insulin (μg/L)						
Week 16	0.87 ± 0.1^a^	0.53 ± 0.08^b^	0.52 ± 0.07^b^	0.34 ± 0.04^c^	0.33 ± 0.03^c^	0.30 ± 0.05^c^

EDA: Extra Domain A; STZ: streptozotocin-treated. For each time point, data sharing the same letter do not differ significantly; ^*^p < 0.05 vs. week 0.
