# Supplementary material for: Lack of Fibronectin Extra Domain A Alternative Splicing Exacerbates Endothelial Dysfunction in Diabetes
Source: Sci Rep. 2016 Nov 29;6:37965. doi: 10.1038/srep37965 (PMC5126581; doi:10.1038/srep37965)
Supplement: Supplementary Information [file srep37965-s1.pdf]

LACK OF FIBRONECTIN EXTRA DOMAIN A ALTERNATIVE SPLICING EXACERBATES ENDOTHELIAL DYSFUNCTION IN DIABETES

[1]Gianluca Gortan Cappellari, [1]Rocco Barazzoni, [1]Luigi Cattin, [2]Andrés F. Muro, [1]Michela Zanetti

[1] Department of Medical, Surgical and Health Sciences, University of Trieste, Trieste, Italy

[2] Mouse Molecular Genetics Laboratory, International Centre for Genetic Engineering and Biotechnology, Trieste, Italy

SUPPLEMENTAL INFORMATION

Supplemental Table 1: Antibodies used in Western blot

Name	Dilution	Product number	Supplier
Total FN	1:2500	F-1509	Sigma Aldrich, St. Louis, MO, USA
eNOS	1:1000	610296	BD Transduction Laboratories, Franklin Lakes, NJ, USA
NOX1	1:250	ab55831	Abcam, Cambridge, UK
NOX4	1:500	ab61248	Abcam, Cambridge, UK
$p22^{phox}$	1:500	ab75941	Abcam, Cambridge, UK
CuZnSOD	1:1000	SOD-101	Stressgen, Ann Arbor, MI, USA
TRAF-6	1:500	04-451	Millipore, Billerica, MA, USA
GTPCH1	1:500	SAB4200046	Sigma Aldrich, St. Louis, MO, USA
GAPDH	1:1000	FL-335	Santa Cruz, Dallas, TX, USA
Anti-Mouse	1:1000	NA931	GE Healthcare Life Sciences, Little Chalfont, UK
Anti-Rabbit	1:1000	7074	Cell Signaling, Beverly, MA, USA
Anti-Rat	1:2000	A9542	Sigma Aldrich, St. Louis, MO, USA

Supplemental Table 2: Primers and probes for Real Time PCR using TaqMan technology (Applied Biosystems).

Gene	RefSeq Accession #	Oligo Sequence
NOX4	NM_015780	Fw. GTT GGG CCT AGG ATT GTG TTT AA<br>Rev AAA GGA TGA GGC TGC AGT TGA<br>Probe FAM - CAG AGC ATC TGC ATC TGT CCT GAA C - TAMRA
p22^{phox}	EU791539	Fw GGT GAG CAG TGG ACT CCC ATT<br>Rev TGC TTG ATG GTG CCT CCA A<br>Probe FAM - AGC CTA AAC CCA AGG AGC GGC CA - TAMRA
TGF-β1	NM_011577	Fw ATC GAC ATG GAG CTG GTG AAA<br>Rev CGA GCC TTA GTT TGG ACA GGA T<br>Probe FAM – AAG CGC ATC GAA GCC ATC CGT G - TAMRA
28S	NR_003279.1	Fw TCG GAA TCC GCT AAG GAG TGT<br>Rev CTC CAG CGC CAT CCA TTT T<br>Probe VIC – CAA CTC ACC TGC CGA ATC AAC TAG CCC T - TAMRA

TaqMan technology probes were synthesized by Applied Biosystems. Primers were synthesized by Eurofins MWG Operon.
